# OCT-NIRS Imaging for Detection of Coronary Plaque Structure and Vulnerability

**DOI:** 10.3389/fcvm.2020.00090

**Published:** 2020-06-04

**Authors:** James Muller, Ryan Madder

**Affiliations:** ^1^Brigham and Women's Hospital, Harvard Medical School, Boston, MA, United States; ^2^Spectrum Health, Grand Rapids, MI, United States

**Keywords:** OCT-NIRS imaging, intravascular coronary imaging, Near-IR Coronary Spectroscopy (NIRS), optical coherence tomography, vulnerable patients and vulnerable plaques, OCT for stenting

## Abstract

A combination optical coherence tomography and near-infrared spectroscopy (OCT-NIRS) coronary imaging system is being developed to improve the care of coronary patients. While stenting has improved, complications continue to occur at the stented site and new events are caused by unrecognized vulnerable plaques. An OCT-NIRS device has potential to improve secondary prevention by optimizing stenting and by identifying vulnerable patients and vulnerable plaques. OCT is already in widespread use world-wide to optimize coronary artery stenting. It provides automated lumen detection and can identify features of coronary plaques not accurately identified by angiography or intravascular ultrasound. The ILUMIEN IV study, to be completed in 2022, will determine if OCT-guided stenting will yield better clinical outcomes than angiographic guidance alone. While the superb spatial resolution of OCT enables the identification of many plaque structural features, the detection by OCT of lipids, an important component of vulnerable plaques, is limited by suboptimal specificity and interobserver agreement. In contrast, NIRS has been extensively validated for lipid-rich plaque detection against the gold-standard of histology and is the only FDA-approved method to identify coronary lipids. Studies in patients have demonstrated that NIRS detects lipid in culprit lesions causing coronary events. In 2019, the positive results of the prospective Lipid-Rich Plaque Study led to FDA approval of NIRS for detection of high-risk plaques and patients. The complementarity of OCT for plaque structure and NIRS for plaque composition led to the sequential performance of NIRS and OCT imaging in patients. NIRS identified lipid while OCT determined the thickness of the cap over the lipid pool. The positive results obtained with OCT and NIRS imaging led to development of a prototype combined OCT-NIRS catheter that can provide co-registered OCT and NIRS data in a single pullback. The data will provide structural and chemical information likely to improve stenting and deliver more accurate identification of vulnerable plaques and vulnerable patients. More precise diagnosis will then lead to OCT-NIRS guided treatment trials to improve secondary prevention. Success in secondary prevention will then facilitate development of improved primary prevention with invasive imaging and effective treatment of patients identified by non-invasive methods.

Despite considerable progress, coronary artery disease continues to be the world's leading cause of death (1). A novel intra-coronary (IC) imaging system that combines optical coherence tomography (OCT) with near-infrared spectroscopy (NIRS) is being developed to improve the secondary prevention of coronary events (2).

The need for a novel instrument is apparent from the continued occurrence of events post-coronary stenting, which is performed approximately 4 million times per year. Complications continue to occur at the stented site, and new events arise from dangerous non-stenotic vulnerable plaques not identified during the initial stenting procedure (Figure 1). In the PROSPECT Study the event rate 3.4 years post-enrollment was 12.9% due to new events caused by the stented site, and 11.6% due to events from non-stenotic vulnerable plaques not identified at the index stenting (3). Recent consensus documents have described the potential for invasive coronary imaging to optimize coronary interventions and improve the treatment of acute coronary syndromes (4, 5).

**Figure 1:**
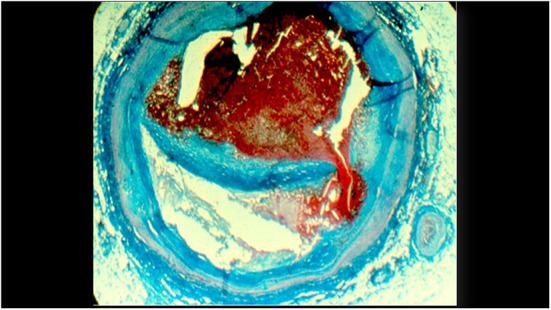
A histologic cross-section of a previously vulnerable coronary plaque causing a fatal myocardial infarction. The cap covering the lipid core ruptured and led to an occlusive thrombus.

The combination OCT-NIRS instrument is being created as a means to improve outcomes post-stenting:

First, by reducing complications at the *stented site*;

Second, by identifying *vulnerable patients* as a means to guide intensity of pharmacologic therapy;

And, third, by identifying *vulnerable plaques* that might benefit from local therapy such as stenting.

While both OCT and NIRS have the potential to contribute to all 3 goals, the primary benefit of OCT is likely to be optimization of the stent result thereby decreasing events at the stented site, while the primary benefit of NIRS is likely to be the identification of vulnerable patients and vulnerable plaques.

The identification of vulnerable plaques, a long-sought goal identified in 1989 (6), and later refined (7), might lead to stenting of dangerous, non-stenotic lesions. This chapter will review the use of OCT and NIRS for the 3 goals of improved stenting, detection of vulnerable patients and detection of vulnerable plaques.

## Experience With Oct

**Figure 2:**
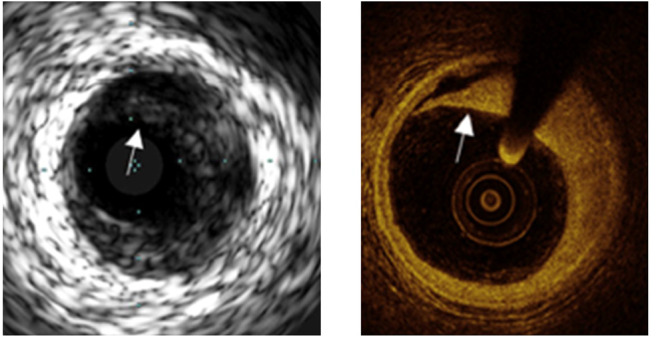
A comparison of images obtained by IVUS and OCT from the same location. The superior resolution of OCT easily permits detection of a dissection (shown by arrows) less apparent by IVUS.

OCT was introduced for medical diagnostics over 20 years ago as a means to use novel laser light sources and fiberoptic catheters to characterize tissue (8, 9). It has been successfully utilized in coronary patients to obtain higher resolution images of plaques and stented lesions than images obtained with intravascular ultrasound (IVUS) (10, 11). The improved resolution of OCT imaging has made it possible to identify many previously hidden features of plaques and stenting [(12), Table 1, Figure 2]. More recently, automated methods have been developed to measure lumen diameter (16).

**Table 1:** Summary of studies evaluating OCT-guided PCI.

**Study**	***N***	**Design**	**Comparison**	**Primary endpoint**	**Major findings**
Prati et al. (13) (CLI-OPCI)	670	Observational	OCT-guided PCI vs. angio-guided PCI	Death or MI at 1 year	OCT-guided PCI associated with lower rate of primary endpoint (adjusted OR 0.49 [95%CI 0.25–0.96], *p* = 0.037)
Meneveau et al. (14) (DOCTORS)	240	Prospective, Randomized	OCT-guided PCI vs. angio-guided PCI	Post-PCI FFR	OCT-guided PCI associated with higher post-PCI FFR (0.94 vs. 0.92, *p* = 0.005)
Ali (15) (ILUMIEN III)	450	Prospective, Randomized	OCT-guided PCI vs. angio-guided PCI vs. IVUS-guided PCI	Post-PCI MSA	OCT-guided PCI achieved non-inferior MSA (5.79 mm^2^) compared to IVUS-guided PCI (5.89 mm^2^; *p* = 0.001 for non-inferiority), but not superior to MSA achieved with angio-guided PCI (5.49 mm^2^; *p* = 0.12)

*Angio, angiography; FFR, fractional flow reserve; IVUS, intravascular ultrasound; MI, myocardial infarction; MSA, minimum stent area; OCT, optical coherence tomography; OR, odds ratio; PCI, percutaneous coronary intervention*.

### OCT for Optimization of Coronary Stenting

Intracoronary structural imaging has shown considerable merit as a means to improve outcomes in patients undergoing percutaneous coronary intervention (PCI). A meta-analysis of 7 randomized trials of IVUS-guided stenting vs. angiographic-guided stenting showed markedly decreased major adverse cardiac events (MACE) rates with IVUS guidance [OR 0.66, 95% CI: 0.52–0.84, *P* = 0.001; (17)]. Although no adequately powered randomized controlled trials have similarly compared OCT-guided stenting vs. angiographic-guided stenting for clinical outcomes, several prior studies evaluating OCT-guided stenting are worth consideration (Table 1). An early observational comparison of OCT-guided PCI vs. angiographic-guided PCI demonstrated improved clinical outcomes, including a reduction in mortality and myocardial infarction, in the OCT-guided cohort (13). These observational findings in favor of OCT have not yet been confirmed in a randomized controlled trial. In the prospective DOCTORS study, patients with non-ST-segment elevation acute coronary syndromes were randomized to undergo OCT-guided PCI or angiography-guided PCI (14). Post-PCI fractional flow reserve (FFR) was significantly higher among patients undergoing OCT-guided PCI. Furthermore, the frequency of an FFR value >0.90 post-PCI was substantially greater with OCT-guidance (82.5%) compared to angiographic guidance (64.2%; *p* = 0.0001).

The ILUMIEN III trial compared OCT-guided, IVUS-guided, and angiographic-guided PCI. The primary endpoint of this trial was minimum stent area (MSA) achieved after PCI, as larger MSA values after stenting have consistently been associated with improved clinical outcomes. OCT-guidance was superior to angiographic-guidance with respect to stent expansion and procedural success. The final minimum stent areas for the 3 approaches were (median [25 percent, 75 percent]) 5.79 [4.54, 7.34] mm^2^ with OCT-guidance, 5.89 [4.67, 7.80] mm^2^ with IVUS-guidance and 5.49 [4.39, 6.59] mm^2^ with angiographic-guidance (15). Since ILUMIEN III was not powered to detect differences in clinical outcomes with the three guidance methods, ILUMIEN IV was launched to compare clinical outcomes in angiographic- vs. OCT-guided stenting in over 3,000 patients undergoing PCI (clinicaltrials.gov NCT03507777). Results of this pivotal trial are expected in 2022.

The use of OCT to guide PCI performance has been made easier by the recent development of software packages providing automated detection of several key anatomic features on the acquired OCT images (18). By automatically detecting lumen profiles, minimum lumen area, metallic stent struts, and stent strut malapposition, these novel software packages can assist operators in planning and optimizing PCI results.

### OCT for Detection of Vulnerable Patients and Vulnerable Plaques

In the prospective PROSPECT Study, IVUS, the structural imaging predecessor of OCT, has been shown to be capable of detecting vulnerable plaques (3). For non-stented sites, IVUS was able to identify plaques likely to cause future events. Non-stenotic sites with a plaque burden ≥70%, minimal lumen area ≤4 mm^2^ and signs of a thin-cap fibroatheroma (TCFA) had a 18.2% chance of causing a major adverse event during 3.4 years of follow-up. The ability of IVUS to identify vulnerable plaques was subsequently confirmed in the VIVA (19) and ATHEROREMO-IVUS studies (20). While the ability to identify plaques at risk was convincingly demonstrated in these studies, some have suggested the positive predictive value achieved by IVUS imaging alone may not be high enough to warrant attempts at local therapy. This is being actively investigated in two ongoing treatment trials of potentially vulnerable plaques, PROSPECT ABSORB (clinicaltrials.gov NCT02171065^1^) and PREVENT (21).

It is not yet known whether OCT is similarly capable of prospectively identifying lesions at risk of future site-specific events. Prati et al. have identified distinct OCT features of culprit lesions in a cross-sectional study (22). Whether non-culprit lesions having these OCT features of vulnerability actually represent vulnerable plaques at risk of causing future events remains unknown. The COMPLETE Study demonstrated that PCI of all stenotic lesions at the time of PCI for a culprit lesion causing a STEMI resulted in improved outcomes. An OCT substudy of 93 patients in the COMPLETE Study found OCT signs indicating that stenotic non-culprit lesions frequently shows signs of vulnerability and suggested that stenting of such lesions might be responsible for the positive results of COMPLETE (23).

**Figure 3:**
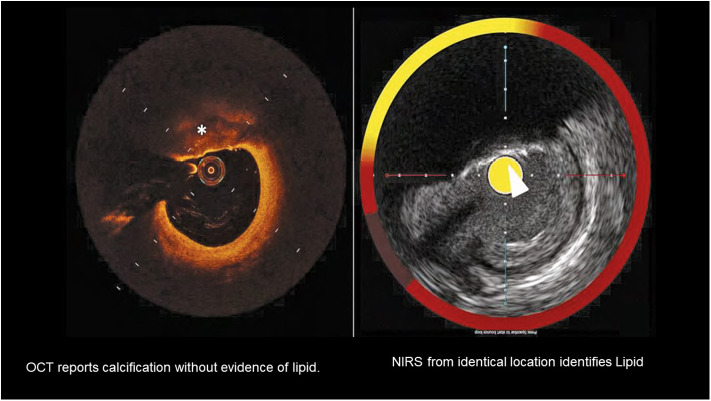
A comparison of OCT detection of lipid with lipid detected by NIRS in the setting of superficial calcification. Calcification is marked by the * on the OCT image and by the arrow on the NIRS-IVUS image. The presence of calcium complicates detection of lipid by OCT.

A major limitation of OCT for detection of vulnerable plaques is the difficulty of identifying lipid deposits in the presence of calcification [(24); Figure 3]. However, OCT can accurately detect more features than IVUS (such as thrombus, vasa vasorum, cholesterol crystals, and cap thickness). Such improved plaque characterization might provide the increased positive predictive value that is needed (25). The prospective CLIMA study has performed baseline OCT imaging in 1,003 patients. The study developed a four component OCT grading system, which includes OCT-identified macrophages (26). The results of the CLIMA study, after 1 year of follow up, have been recently reported and demonstrated the presence of MLA <3.5 mm^2^, fibrous cap thickness <75 μm, lipid arc >180°, and macrophages, as detected by OCT, were associated with an increased risk of the primary endpoint, a composite of death and target segment myocardial infarction (27). The simultaneous presence of all four of these OCT characteristics was an independent predictor of the primary endpoint with a HR of 7.54 (95% CI 3.1–18.6).

A vulnerable plaque *treatment* trial based on OCT-guidance has also been initiated. In the PREVENT Study Park et al. have enrolled over 1,000 patients with OCT, NIRS or IVUS signs of a non-stenotic vulnerable plaque (21). The suspected vulnerable plaques have been randomized to optimal medical therapy (OMT) alone, or OMT plus a stent or scaffold. Results are expected in 2022.

Whereas, the ability of OCT to prospectively detect vulnerable plaques has not yet been proven, OCT has been shown to be capable of identifying vulnerable patients, defined as those at an increased risk of future patient-level cardiovascular events (Table 2). In an observational study of 1,474 patients undergoing OCT imaging at baseline, the presence of non-culprit LRP detected by OCT in the target vessel was associated with a higher risk of patient-level major adverse cardiovascular events during 48 months of follow up (28). The results of the CLIMA study described above also support the ability of OCT to detect vulnerable patients (27).

**Table 2:** Summary of OCT and NIRS studies for the detection of vulnerable patients.

**References**	***N***	**Modality**	**Primary endpoint**	**Follow up**	**Major findings**
Xing et al. (28)	1,474	OCT	MACE (cardiac death, acute MI, ischemia-driven revasc)	4 year	Non-culprit LRP in target vessel associated with increased risk of MACE RR 2.06 (95% CI 1.05–4.04)
Prati et al. (27)	1,003	OCT	Death and target segment MI	1 year	Simultaneous presence of 4 OCT features (MLA <3.5 mm^2^, fibrous cap thickness <75 μm, lipid arc >180°, macrophages) associated with primary endpoint: HR 7.54 (95% CI 3.1–18.6)
Oemrawsingh et al. (29)	203	NIRS	MACE (all-cause mortality, non-fatal ACS, stroke, unplanned coronary revasc)	1 year	LCBI in non-culprit coronary artery at or above median of 43 for study population associated with primary endpoint: HR 4.04 (95% CI 1.33–12.29)
Madder et al. (30)	121	NIRS	MACE (all-cause mortality, non-fatal ACS, cerebrovasc events)	≥1 year	MaxLCBI_4mm_ ≥400 in non-stented segments of target artery associated with primary endpoint: HR 10.2 (95% CI 3.4–30.6)
Danek et al. (31)	239	NIRS	MACE (cardiac mortality, ACS, stroke, unplanned revasc)	5 years	Non-target vessel LCBI ≥77 associated with primary endpoint: HR 14.04 (95% CI 2.47–133.51)
Schuurman et al. (32)	275	NIRS	MACE (all-cause death, non-fatal ACS, unplanned revasc)	4 years	Each 100 unit increase maxLCBI_4mm_ in non-culprit artery associated with primary endpoint: HR 1.19 (95% CI 1.07–1.32)
Karlsson et al. (33)	144	NIRS	MACE (all-cause mortality, ACS requiring revasc, cerebrovasc events)	≥1 year	MaxLCBI_4mm_ ≥400 in non-culprit segments of culprit artery associated with primary endpoint: HR 3.67 (95% CI 1.46–9.23)
Waksman et al. (34)	1,563	NIRS	MACE (cardiac death, cardiac arrest, non-fatal MI, ACS, revasc, readmission for angina with more than 20% diameter stenosis progression)	2 years	Each 100 unit increase in non-culprit maxLCBI_4mm_ associated with primary endpoint: HR 1.18 (95% CI 1.05–1.32)

*ACS, acute coronary syndrome; cerebrovasc, cerebrovascular; HR, hazard ratio; MACE, major adverse cardiovascular events; MI, myocardial infarction; mo, months; NIRS, near-infrared spectroscopy; OCT, optical coherence tomography; revasc, revascularization; RR, risk ratio*.

## Experience With NIRS

**Figure 4:**
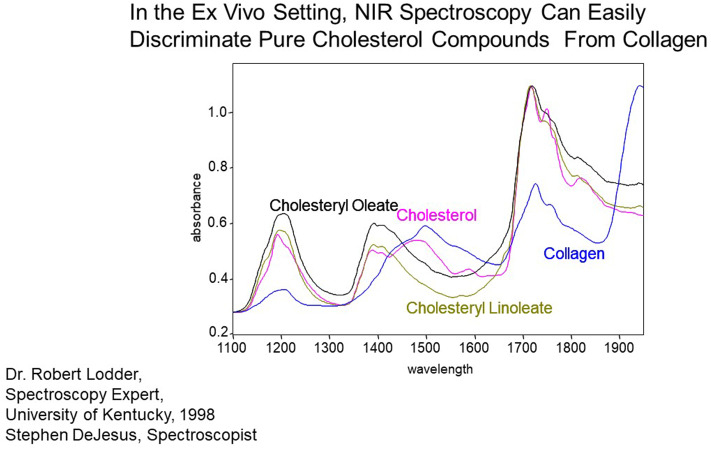
Measurement of pure chemicals by spectroscopy in the absence of blood flow and motion. Substances of interest, such as collagen and cholesterol, are easily identified by their variable absorbance at different near-IR wavelengths.

Intracoronary NIRS imaging was developed as a means to detect the lipid-core plaques suspected to be vulnerable to disruption and thrombosis (Figure 4). Initial studies demonstrated that NIRS could detect lipid core plaques in human coronary autopsy specimens as documented by histology (35). In 2019 the U.S. FDA cleared NIRS for the detection of patients and plaques at high-risk of causing a MACE on the basis of the Lipid-Rich Plaque Study results (34).

### NIRS for Optimization of Coronary Stenting

It has been observed that dilation of a lipid-rich plaque is associated with no-reflow and periprocedural myocardial infarction, a serious complication of PCI. Using data from a large NIRS registry, Goldstein *et al* reported target lesions with a maxLCBI_4mm_ ≥ 500 were associated with periprocedural myocardial infarction during PCI in 50% cases (36). This led to the concept that the use of a filter might be helpful in preventing peri-stenting myocardial infarction. The CANARY study confirmed the association between large LRP detected by NIRS and the risk of periprocedural myocardial infarction (37). However the CANARY Study was unable to demonstrate a beneficial effect of distal embolic protection (37). A recent report indicates that stenting of plaques with increased lipid by NIRS is associated with signs of microvascular injury (38).

The COLOR Registry demonstrated that stenting of lipid-rich plaque, as detected by NIRS carried no increased longer term risk, which supports study of the use of stenting for vulnerable plaques (39). It is possible that NIRS may identify optimal non-lipid-rich landing zones for the ends of stents and guide the selection of stent length to ensure adequate coverage of LRP. In this regard, a prior study has demonstrated LRP to frequently extend beyond the angiographic margins of the target lesion (40). Furthermore, LRP in the stent margins, as identified by OCT, has been associated with an increased risk of subsequent restenosis, with larger lipid cores seemingly carrying greater risk (41). Whether LRP identified by NIRS in the stent margins after PCI similarly identifies a greater risk of restenosis has not been adequately investigated.

### NIRS for Detection of Vulnerable Patients and Vulnerable Plaques

Multiple studies have demonstrated that the detection by NIRS of lipid in the walls of the coronary arteries identifies vulnerable patients at increased risk of a new event (33). There is also evidence that NIRS can detect vulnerable plaques, a more difficult goal than identifying vulnerable patients, but one that could lead to effective local therapy. The initial data suggesting that NIRS could identify vulnerable plaques were obtained from cross-sectional studies, in which NIRS evidence of a lipid core plaque was found at the site of culprit lesions causing ST-segment elevation myocardial infarction (STEMI) (42, 43). More recently Terada et al. demonstrated that the NIRS-IVUS instrument (Infraredx, Inc., a Nipro Company) can differentiate plaque rupture from erosion from calcified nodule as the cause of a coronary event (44).

The promising cross-sectional studies have now been supplemented with the required prospective data. As mentioned above, in April 2019, the US FDA approved NIRS for detection of both vulnerable patients and vulnerable plaques on the basis of the results of the Lipid-Rich Plaque Study (34). Thirty mm segments of artery were designated as “Ware Segments” and assessed for lipid core plaque by NIRS imaging at baseline. Lipid content was designated as the maximal lipid-core burden index in 4 mm lengths of pullback (maxLCBI_4mm_). Patients were then followed for 2 years for evidence of new coronary events. For segments with a maxLCBI_4mm_ more than 400, the unadjusted hazard ratio (HR) for non-Culprit-MACE was 4.22 (2.39–7.45; *p* < 0·0001) and the adjusted HR was 3.39 (1.85–6.20; *p* < 0·0001).

While an adjusted HR of 3.39 is significant, a higher hazard ratio would be helpful for identification of plaques that might benefit from local therapy.

Results of a second study of NIRS-IVUS imaging to detect vulnerable plaque and vulnerable patients are expected in 2020. The PROSPECT 2-ABSORB (P2A) study, which is being led by Drs. David Erlinge and Gregg Stone, has completed enrollment of 900 patients in Sweden, Norway and Denmark (Clinicaltrials.gov NCT02171065). Among the endpoints to be evaluated will be the ability of additional information provided by IVUS to increase the hazard ratio for vulnerable plaques detected by NIRS. The ABSORB portion of P2A is a pilot study in 200 patients of the treatment of non-stenotic vulnerable plaques randomized to OMT or OMT plus a local therapy with a bioresorbable vascular scaffold (BVS). The results of this study will have to be interpreted in the context of the known scaffold thrombosis risk associated with BVS.

## Studies With Both Oct and NIRS Measurements

The promising results cited above with both OCT and NIRS have led to interest in imaging with both OCT and NIRS in patients undergoing PCI. These studies were performed with separate OCT and NIRS catheters, which necessitated two pullbacks, and required efforts to establish co-registration of the OCT and NIRS images. Bourantas et al. have reviewed the overall status of hybrid instruments that can provide multiple types of information about a plaque in a single pullback (45).

For OCT plus NIRS imaging, the combination was successful in two separate reports in identifying thin caps over neoatheroma formed inside stents (46, 47). These results suggest combined OCT-NIRS imaging in pre-existing stents may overcome some of the limitations inherent to NIRS-IVUS imaging in identifying neoatherosclerosis (48).

**Figure 5:**
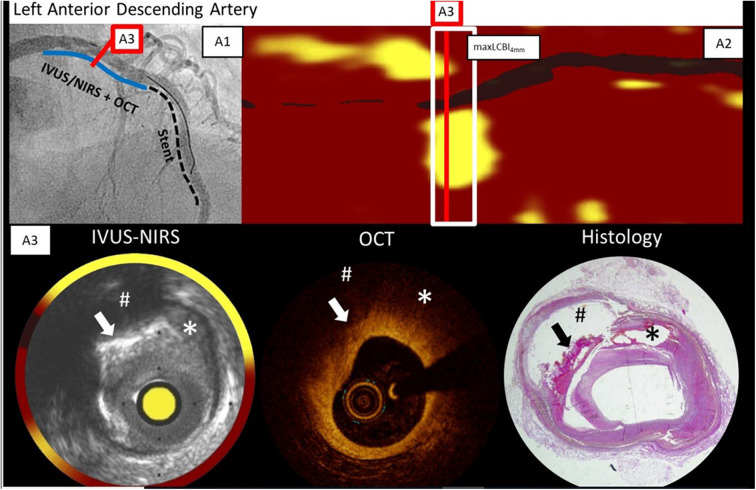
Angiogram, NIRS, OCT, and histology findings in a patient who died of ventricular rupture 5 days after imaging. The blue line on the angiogram (A1) shows the IVUS-NIRS and OCT pullback location in LAD during stenting at a more distal location. The chemogram (A2) and cross-section IVUS-NIRS (A3) shows lipid from 10 p.m. to 3 p.m. OCT from the same cross-section shows a thick cap of 300 microns, and signs of calcification which complicate the detection of lipid by OCT. In each image, the arrow marks the location of superficial calcium, the hash-tag marks the location of a lipid core underlying the calcification, and the the asterisk marks the location of a lipid pool. Adapted from Zanchin et al. (49). The findings show the complementarity of NIRS and OCT data—NIRS identifies lipid without interference by calcium and OCT shows the thickness of the cap over the lipid. In this case in which the plaque was not causing obstruction, the lipid core would not be expected to be dangerous since a thick cap is present.

Räber et al. performed NIRS and OCT imaging in a patient with a myocardial infarction who unfortunately died 5 days later of a ventricular rupture [(5); Figure 5]. This permitted comparison of OCT and NIRS data with histology (49). *In vivo* NIRS showed a large lipid-rich plaque validated by histology as expected from prior *ex vivo* autopsy validation studies. The *in vivo* OCT image indicated that the lipid core was covered by a thick cap—a finding also validated by histology. Hence the NIRS plus OCT suggested that a large lipid core was present, but that it was less likely to be vulnerable in the near-term due to the presence of thick cap.

**Figure 6:**
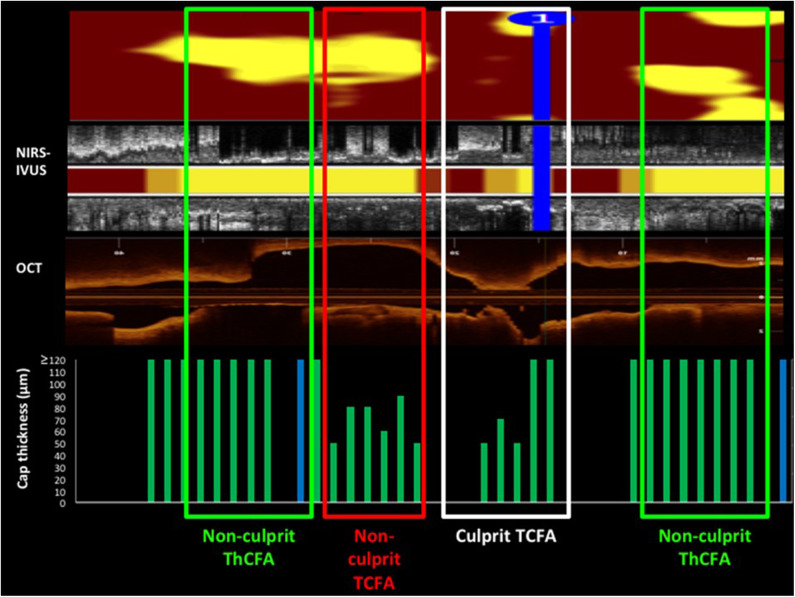
A comparison of NIRS-IVUS and OCT imaging data in a patient with a coronary event. OCT was used to measure cap thickness in regions with lipid detected by NIRS and was able to differentiate lipid cores with thick and thin caps. The culprit lesion, identified by angiography plus IVUS and OCT imaging is indicated on the NIRS-IVUS and OCT pullbacks. The culprit lesion occurred at the relatively small lipid core plaque that had a thin cap as determined by OCT. The NIRS chemogram shows 2 large lipid core plaques (yellow spots) at non-culprit sites. Personal Communication from Dr. Ryan Madder.

Madder obtained OCT and NIRS data in a patient who experienced a coronary event [(50); Figure 6]. The NIRS chemogram showed 2 large and one small lipid core plaques. The OCT showed that the culprit event was caused not by the large lipid core plaques that had thick caps but by the smaller lipid core plaque that had a thin cap.

## Development of a Combination Oct-NIRS Catheter

**Figure 7:**
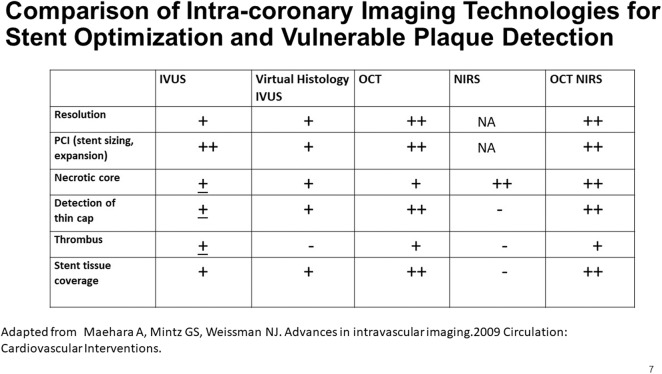
A comparison of the capabilities of multiple imaging modalities for the detection of various features of interest in a coronary artery. Adapted from Maehara et al. (51).

The complementarity of OCT and NIRS data led to interest in a combination OCT-NIRS catheter. The hybrid catheter would permit collection of automatically co-registered data in a single pullback. Features of a combination OCT-NIRS device are shown in Figure 7. OCT provides a broad range of features, while the sole contribution of the NIRS is the accurate and automated detection of lipid core plaque.

**Figure 8:**
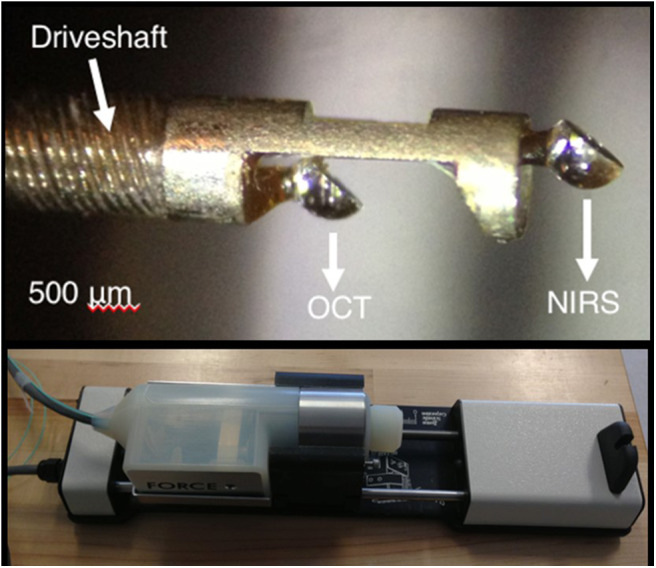
A close-up of the tip of the prototype OCT-NIRS catheter created by the Tearney Lab. Light travels down a first optical fiber, and returns to provide the NIRS signal in a second optical fiber.

**Figure 9:**
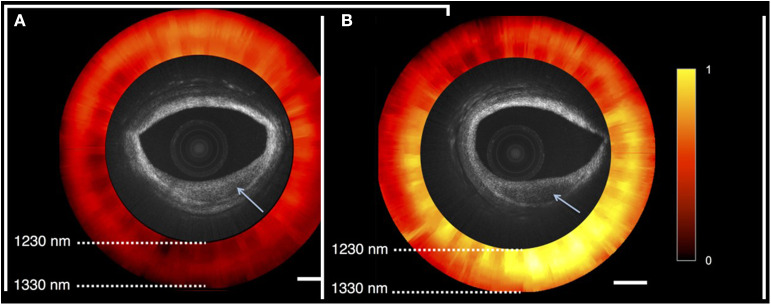
OCT-NIRS images of cadaver coronary artery. Both OCT images show lesions with reduced backscattering (arrow). NIRS image (red and yellow) shows absorption spectra compatible with fibrotic tissue (red) in the left image **(A)**, and lipid-rich tissue (yellow) in the right image **(B)**. As shown on the right image, a promising feature of the combined OCT-NIRS device is that OCT may facilitate determining the depth of lipid cores identified by NIRS.

A prototype functional OCT-NIRS catheter has been developed in the Tearney Lab at the Massachusetts General Hospital, Boston MA [(2); Figure 8]. Studies performed in autopsy specimens demonstrated the detection of lipid by spectroscopy and structural features, including cap thickness, by OCT (Figure 9). As illustrated in Figure 9, a promising feature of the combined OCT-NIRS device is that OCT may facilitate determining the depth of lipid cores identified by NIRS. SpectraWAVE, Inc. is now building a commercial, combination OCT-NIRS catheter that will be available for clinical use in 2021.

## Conclusion

OCT already provides valuable information for PCI procedures, and NIRS has already been demonstrated to identify vulnerable patients based on the detection and quantification of coronary lipid. However, the effort to identify vulnerable plaques which has been in progress for 30 years and has yet to provide a validated, widely used method to identify and treat the focal lesions. This has led to criticism of the search for the vulnerable plaque, and the admonition that efforts should be shifted to identification of the vulnerable patient for this systemic disease (52). A counter-argument can be made in favor of identifying both the vulnerable patient, and the most dangerous plaques that are the focal manifestation of the disease.

It is expected that the OCT-NIRS catheter, with automated co-registration of data, will contribute to the triple goal of improving stenting and detecting and treating vulnerable patients and vulnerable plaques. An added benefit of improved secondary prevention will be an impetus for improved primary prevention made possible by precise invasive imaging and treatment of those deemed to be at higher risk by non-invasive imaging (53, 54).

## Author Contributions

All authors listed have made a substantial, direct and intellectual contribution to the work, and approved it for publication.

## Conflict of Interest

JM is CMO of SpectraWAVE, Inc, which is building an OCT-NIRS coronary catheter. RM has received research support and speaker honoraria from Infraredx and serves on the advisory board of Spectra WAVE, Inc.

## Footnotes

^1^ClinicalTrials.gov NCT02171065, PROSPECT2-ABSORB Study.
